# Preventing *Candida albicans* from subverting host plasminogen for invasive infection treatment

**DOI:** 10.1080/22221751.2020.1840927

**Published:** 2020-11-03

**Authors:** Si-Min Chen, Zui Zou, Shi-Yu Guo, Wei-Tong Hou, Xi-Ran Qiu, Yu Zhang, Li-Jun Song, Xin-Yu Hu, Yuan-Ying Jiang, Hui Shen, Mao-Mao An

**Affiliations:** a Department of Pharmacology, Shanghai Tenth People's Hospital, Tongji University School of Medicine, Shanghai, People’s Republic of China; b Department of Anesthesiology, Changzheng Hospital, Second Military Medical University, Shanghai, People’s Republic of China; c Department of Laboratory Diagnosis, Shanghai East Hospital, Tongji University School of Medicine, Shanghai, People’s Republic of China

**Keywords:** *C. albicans*, Enolase, fungal infection, plasminogen, invasive candidiasis

## Abstract

*Candida albicans* is a common fungal pathogen in humans that colonizes the skin and mucosal surfaces of the majority healthy individuals. How *C. albicans* disseminates into the bloodstream and causes life-threatening systemic infections in immunocompromised patients remains unclear. Plasminogen system activation can degrade a variety of structural proteins *in vivo* and is involved in several homeostatic processes. Here, for the first time, we characterized that *C. albicans* could capture and “subvert” host plasminogen to invade host epithelial cell surface barriers through cell-wall localized Eno1 protein. We found that the “subverted” plasminogen system plays an important role in development of invasive infection caused by *C. albicans* in mice. Base on this finding, we discovered a mouse monoclonal antibody (mAb) 12D9 targeting *C. albicans* Eno1, with high affinity to the _254_FYKDGKYDL_262_ motif in α-helices 6, β-sheet 6 (H6S6) loop and direct blocking activity for *C. albicans* capture host plasminogen. mAb 12D9 could prevent *C. albicans* from invading human epithelial and endothelial cells, and displayed antifungal activity and synergistic effect with anidulafungin or fluconazole in proof-of-concept *in vivo* studies, suggesting that blocking the function of cell surface Eno1 was effective for controlling invasive infection caused by *Candida* spp. In summary, our study provides the evidence of *C. albicans* invading host by “subverting” plasminogen system, suggesting a potential novel treatment strategy for invasive fungal infections.

## Introduction

*Candida albicans* is a common fungal microorganism that colonizes the oral, genital and gastrointestinal surfaces in most healthy individuals [1,2]. Colony maintenance requires a delicate balance between fungal proliferation and host immune recognition [3]. In immunocompromised patients, *C. albicans* may disseminate into the bloodstream and lead to life-threatening systemic candidiasis [4,5]. However, anticandidal therapy is often of limited effectiveness in these patients, resulting in unacceptably high rates of mortality and morbidity. The associated mortality rate for systemic infection is reported to be greater than 30%, highlighting the potential critical impact of *C. albicans* on global health burden [1,6]. There is a justifiable need for more research to develop novel, more efficacious antifungal treatments.

In addition to host immunological defences, physical barriers also exist between different tissues to prevent dissemination of microorganisms [7]. It takes several steps for *C. albicans* to establish invasive infection, including damage and invasion of the mucosal epithelium, vascular dissemination and seeding of yeast cells into the bloodstream, and target tissue invasion and colonization. The key for *C. albicans* to establish an invasive infection is damage and invasion of epithelial or endothelial cells [8,9]. Therefore, blocking this process might be an attractive approach for *C. albicans* caused invasive infections. Cell wall, the outermost cellular structure of *C. albicans*, is composed of cross-linked polysaccharides and glycoproteins, which are not only critical for the integrity and shape of the fungal cells as they grow and differentiate, but also a weapon for invading host [8]. The exact mechanism of *C. albicans* cell wall damaging the host physical barriers has not been well characterized.

The physiological function of plasmin is integral to various homeostatic processes including blood coagulation, cell migration, and tissue and wound repair [10]. The extracellular matrix (ECM) is the acellular protein component of animal tissues. It defines the basement membrane (BM) region and constitutes an anchoring platform for the epithelium, and is a part of the connective tissues that fill the interstitial spaces in the tissue parenchyma [11,12]. The ECM is basically composed of fibronectin (Fn), laminin (Ln), and collagen. Plasminogen is synthesized in the liver and released into the bloodstream as a zymogen with high concentrations (around 180 mg/mL) [13]. The zymogen is converted into active plasmin protease by tissue-type plasminogen activator (tPA) and urokinase plasminogen activator (uPA) [11]. Plasmin is a broad-specificity serine protease that degrades Fn, Ln, collagen, and a variety of other structural proteins. Plasmin can also activate other proteolytic enzymes, such as matrix metalloproteinases (MMPs), which act to degrade the tight junction components of microvascular endothelial cells [13]. It has been reported that some invasive pathogens, such as *Staphylococcus aureus*, *Streptococcus pneumoniae* and *Haemophilus influenza*, can associate with host plasminogen to their surface, and then converted plasminogen into active plasmin for degrading host ECM proteins and cell barriers [13–16]. This mechanism is known to allow pathogens to migrate from periodontal tissue to various host organs via bloodstream [11]. Furthermore, several fungal pathogens including *C. albicans*, *A. fumigatus* and *C. neoformans* are also known to be able to bind plasminogen [17–19]. However, the molecular mechanism of fungal cell walls interacting with host plasminogen and the role of this interaction in establishment of invasive infections have not been elucidated.

Enolase is a dimeric enzyme that catalyses the penultimate step in glycolysis, interconverting 2-phosphoglycerate (2-PGA) and phosphoenolpyruvate (PEP) in cytoplasm [20,21]. Interestingly, localization of enolase to cell surface has been observed in several bacteria such as *Borrelia burgdorferi*, *Staphylococcus aureus*, *Group A streptococci* and *Listeria monocytogenes* [22–25]. *C. albicans* cell surface located Eno1 has been identified as a moon-lighting protein with unrelated glycolytic enzyme function such as transglutaminase activity and major antigen in patients with candidiasis [20,21,26]. Previous studies have demonstrated *C. albicans* Eno1 involved in colonization of mammalian intestinal epithelium and invasion of human brain microvascular endothelial cells *in vitro* [27,28]. In addition, Eno1 null mutant *C. albicans* exhibited avirulent in animal [29]. However, the mechanism of Eno1 involved in the virulence of *C. albicans* remains unclear.

In the present study, we first characterized that *C. albicans* cell wall-localized Eno1 could capture and “subvert” host plasminogen for facilitating invasive infection. We further described a mouse monoclonal antibody (mAb) 12D9 targeting *C. albicans* Eno1, with a direct ability to block *C. albicans* capturing host plasminogen. Finally, we conducted proof-of-concept *in vivo* studies and showed that neutralization of cell surface Eno1 was effective for controlling *C. albicans* caused invasive infection.

## Materials and methods

### Ethics statement

All animal experiments were performed using procedures outlined by the “Regulations on the Administration of Laboratory Animals” approved by the State Council of the People's Republic of China. The animal experiment protocol has been verified and approved by the Animal Care and Use Committee of Tongji University (TJCAC-018-033).

### Mice

Female C57BL/6 and BALB/c mice (6–8 weeks old) were obtained from Shanghai Laboratory Animal Center (SLAC) of the Chinese Academy of Sciences (Shanghai, China).

### Reagents, antibodies and plasmids

IPTG (isopropyl-β-D-thiogalactopyranoside) and DTT were purchased from Sangon Biotech. Ni-nitrilotriacetic acid (Ni-NTA) was purchased from QIAGEN. Human plasminogen, ϵ-aminocaproic acid (ϵ-ACA), Creatinine Assay Kit, glass beads, urokinase-type plasminogen activator, anidulafungin, fluconazole, and DMEM medium were purchased from Sigma-Aldrich. Human Endothelial Serum Free Medium, HuMEC Basal Serum-Free Medium and Blood Urea Nitrogen Detection Kit were purchased from Thermo Fisher Scientific. Mouse nonspecific IgG2a was obtained from Invivogen. Cy3-labelled secondary antibody was purchased from Invitrogen. Chromogenic substrate D-Val-Leu-Lys-pNA·2HCl was purchased from Innovative Research. The LDH Cytotoxicity Assay Kit was obtained from Beyotime. PrimeScript ^TM^ RT Reagent Kit and the PrimeSTAR® Max DNA Polymerase were obtained from TaKaRa Bio. Rabbit anti-plasminogen antibody was obtained from Acris Antibodies. Anti-actin monoclonal antibody, and horseradish peroxidase (HRP)-conjugated anti-rabbit IgG were obtained from Abcam. HRP-labelled goat anti-mouse antibody was purchased from Dingguochangsheng Biotechnology. pET-21a (+) was purchased from Novagen.

### Candida spp. strains growth conditions

All strains were maintained on SDA agar plates (1% peptone, 4% dextrose, and 1.8% agar) and grown in YPD broth (1% yeast extract, 2% peptone, and 2% dextrose) at 30°C. *C. albicans* SC5314 was kindly provided by Sanglard D (Centre Hospitalier Universitaire Vaudois). Clinical isolates *C. albicans* 0304103 and Y0109 were kindly provided by Dr. Jun Gu (Changhai Hospital, Shanghai, China). *C. glabrata* ATCC28226, *C. krusei* ATCC 6258, *C. parapsilosis* ATCC 34136 and *C. tropical* ATCC 20026 were obtained from ATCC.

For hyphal growth, exponentially growing *C. albicans* yeast cells were washed in PBS buffer and cultured in RPMI 1640 medium at 37°C for 3 h.

To obtain growth curve, exponentially growing *C*. *albicans* SC5314 were washed and resuspended in fresh YPD broth [optical density (OD)620 = 0.1] at 30°C with 200 rpm shaking, then the OD_620_ value was determined at the indicated time points.

### Plasminogen-binding assay

Recombinant *C. albicans* Eno1 or other cell wall proteins (0.5 μg) were coated in 96-well plates (Nunc-Immuno) and blocked with PBS containing 5% bovine serum albumin (BSA). Then human plasminogen (0.5 μg per well) or negative control PBS buffer was added and incubated at 37°C for 1 h. Plasminogen binding was detected using a rabbit anti-plasminogen antibody (Acris Antibodies) and HRP-conjugated anti-rabbit IgG (Abcam). The reaction was quenched by 1 M H_2_SO_4_. Then the absorbance was measured at 450 nm using a multi-mode microplate reader.

For plasminogen binding to *C. albicans* assay, human plasminogen was labelled with allophycocyanin 650 according to the manufacturer’s instructions (Dojindo Molecular Technologies). Yeast (1 × 10^7^ cells) or hyphal form (1 × 10^6^ cells) of *C. albicans* were incubated in PBS containing allophycocyanin 650-labelled human plasminogen (5 μg/mL) at 37°C for 30 min. Then the binding of plasminogen with *C. albicans* was observed using a laser scanning confocal microscope (excitation wavelength, 650 nm; emission wavelength, 660 nm) (TCS SP5; Leica).

### Plasminogen activity assay

The chromogenic substrate D-Val-Leu-Lys-pNA·2 HCl (Innovative Research) was dissolved in buffer (75 mM Tris HCl, 318 mM NaCl, pH 7.5) (0.25 mM), and treated with human plasminogen in the presence of *C. albicans*, non-*albicans Candida spp*. or other recombinant expressed *C. albicans* cell wall proteins. Urokinase-type plasminogen activator (0.1 g/mL) (Sigma-Aldrich) was used as positive control. The reaction mixture was incubated at 37°C for 24 h and then the absorbance was measured at 405 nm using a multi-mode microplate reader.

### Host cell damage assay

Human Umbilical Vein Endothelial Cells (HUVECs) and Caco-2 intestinal epithelial cells were cultured with Human Endothelial Serum Free Medium and HuMEC Basal Serum-Free Medium in 96-well plate (1 × 10^4^ cells per well) at 37°C with 5% CO_2_, respectively. Then *C. albicans* (2.5 × 10^3^ cells per well) and Eno1 mAb or negative control mouse nonspecific IgG2a were added to the 96-well plates. After 12 h co-culture, cell supernatant was transferred for lactate dehydrogenase (LDH) assay using commercial kit (Beyotime). Maximal LDH release was determined by adding an LDH releasing agent (Beyotime) in parallel. Relative LDH release was measured as follows: LDH (%) = (OD_490_ indicated cells – OD_490_ control)/ (OD_490_ maximal LDH release – OD_490_ control).

### Murine systemic candidiasis model

For the *Candida* spp. caused systemic infection *in vivo*, 6–8 weeks old C57BL/6 female mice were intravenously injected with 200 μl of PBS buffer containing indicated live *Candida* spp. cells [1 × 10^6^ Colony Forming Unit (CFU) per mouse] for *C. albicans*, (*C. tropicalis and C. parapsilosis*; 1 × 10^7^ CFU per mouse for *C. glabrata*; 5 × 10^5^ CFU per mouse for *C. krusei*.)*.* Then the survival was monitored for 30 days. And the kidneys and liver were removed 2 days post-infection and then homogenized in PBS buffer to determine fungal burden or fixed in 10% neutral formalin for haematoxylin and eosin (H&E) or periodic acid-schiffs (PAS) staining. Renal function tests were carried out using the Blood Urea Nitrogen Detection Kit (Thermo Fisher Scientific) and Creatinine Assay Kit (Sigma-Aldrich). To abolish plasminogen activity *in vivo*, mice were intraperitoneally injected with *ϵ*-ACA (30 mg/kg) twice daily for 5 days.

### Western blot

*C. albicans* cells were collected and then resuspended in ice-cold lysis buffer (150 mM NaCl, 50 mM Tris-HCl, 1 mM DTT, 0.5 mM PMSF, and 5 mg/mL of the protease inhibitors leupeptin, pepstatin, and antipain, pH 7.4). The cells were then lysed with glass beads (0.40 mm in diameter, Sigma-Aldrich) in a cell homogenizer (Braun, MSK). The insoluble fraction containing cell wall components was harvested by centrifuging at 8,000 g; the pellet was then washed with ice-cold water and boiled with extraction buffer (50 mM Tris-HCl, pH 7.5, 0.1 M EDTA, 2% SDS) to obtain cell wall proteins for immunoblot analysis. The cell wall proteins were subjected to SDS-PAGE, blotted with the Eno1 immune serum from mice (1:1000 dilution rate) and secondary antibodies, and then developed with the chemiluminescence method according to the manufacturer’s instructions (Millipore) using the ECL detection system (GE Healthcare). The densitometry of indicated blot was quantified using Image J software (National Institutes of Health, USA).

### Quantitative real-time PCR

HUVECs or Caco-2 intestinal epithelial cells (2 × 10^7^ cells) were challenged by exponentially growing *C. albicans* (MOI = 1) for 1 or and 3 h respectively. Then the total mRNA of *C. albicans* SC5314 was extracted using the PureLink™ RNA Mini Kit (Invitrogen) according to the manufacturer’s protocol. RT–PCR analysis was performed using the PrimeScript ^TM^ RT Reagent Kit (TaKaRa Bio). The individual gene-specific primers are listed in Table S1.

### Expression and purification of C. albicans cell wall proteins

The gene encoding *C. albicans* Eno1 or its different domain, and other cell wall proteins were amplified from *C. albicans* SC5314 genomic DNA (the primers are listed in Table S2) and cloned into pET-21a (+) containing 6×His-tag. And then the plasmids were transformed into BL21 (DE3) pLysS cells for protein expression. The transformants were cultured overnight at 37°C and diluted 1:100 in fresh LB culture. When the medium OD_600_ was up to 0.6 at 37°C, IPTG was added at a final concentration of 0.1 mM and the transformants were grown at 16°C for 16 h. After that the transformants were lysed by sonication, and the target protein was purified by Ni-NTA (Qiagen).

### Generation and selection of monoclonal antibodies against C. albicans Eno1

BALB/c mice (6–8 weeks old) were immunized with *C. albicans* Eno1 combined with complete Freund’s adjuvant or incomplete Freund’s adjuvant at multiple sites. Spleens from immunized mice were collected and underwent fusion with P3X63Ag8.653 myeloma cells to generate B-cell hybridomas [30]. Hybridoma supernatants were screened for activity of binding with Eno1 using ELISA method and inhibiting *C. albicans* Eno1 binding to human plasminogen. The most active hybridomas were subjected to limiting dilution to obtain mAb. As a result, mAb 12D9 was selected for further study.

### Enzyme-linked immunosorbent assay (ELISA)

Recombinant *C. albicans* or non-*albicans Candida* spp. Eno1, indicated domains or mutants of *C. albicans* Eno1 were coated in 96-wells plates (Thermo Scientific, 442404) (0.25 μg/well) overnight and blocked with PBS containing 5% BSA. The plates were then subjected to a series dilution of anti-Eno1 antibody and incubated overnight. After incubation with an HRP-labelled goat anti-mouse antibody (Dingguochangsheng Biotechnology) for 1 h, TMB substrate was added and incubated for 15 min incubation, and then 1 M H2SO4 were added to stop the reaction. Finally, the absorbance of reaction mixture was measured at 450 nm using a multi-mode microplate reader. 96-well microplate coated with Bovine Serum Albumin (BSA) was applied as parallel control in the analysis of plasminogen binding to recombinant *C. albicans* proteins. The test data of parallel control was deducted to eliminate the non-specific binding of plasminogen or antibodies to the microplate.

### Surface plasmon resonance (SPR) analysis

Biacore T200 instrument (GE Healthcare) combined with a CM5 sensor chip (GE Healthcare) was used for SPR analysis. *C. albicans* Eno1 protein was immobilized in parallel-flow channel on a BIAcore™ CM5 sensor chip using the Amine Coupling Kit (GE Healthcare). Serial dilutions of mAb 12D9 were injected into the flow system. Experiments were conducted using PBS as running buffer, and the analyte was injected at a flow rate of 30 μl/min. The association time was 90 s and the dissociation time was 60 s. The affinity constants for binding were obtained using BIA evaluation software and a 1:1 Langmuir binding model.

### Confocal laser scanning microscopy and flow cytometry analysis

Exponentially growing *C. albicans* SC5314 cells were incubated with mAb 12D9 or negative control mouse nonspecific IgG2a at 16°C overnight. Then the *C. albicans* SC5314 cells were washed with PBS and incubated with a Cy3-labelled secondary antibody (Invitrogen) at 30°C for 1 h. The stained *C. albicans* SC5314 cells were then mounted to microscope slides and analysed with a confocal laser scanning microscope (TCS SP5; Leica). For flow cytometry analysis, the stained cells were fixed with 1% formaldehyde overnight and analysed by flow cytometry (BD FACSVerse).

### mAb opsono-phagocytic and killing assay

Thioglycollate-elicited peritoneal macrophages and neutrophils were isolated as previously described [31]. For phagocytosis assay, exponentially growing *C. albicans* SC5314 cells were co-cultured with peritoneal macrophages (1 × 10^6^ cells) (MOI = 0.4) in the presence of indicated concentrations of m Ab 12D9 at 37°C for 1 h. Then the unbound *C. albicans* SC5314 were removed by three washes of PBS buffer. Then the mixture was plated on SDA agar and incubated at 30°C for 48 h and the live *C. albicans* were counted.

For neutrophils killing assay, exponentially growing *C. albicans* SC5314 (1 × 104 cells) were incubated with indicated concentrations of mAb 12D9 at 30°C for 1 h. Then the *C. albicans* cells were collected and co-cultured with peritoneal neutrophils (2 × 10^6^ cells) with MOI = 0.05 at 4°C for 1 h to make the cells settle, and then transferred to 37°C for another 1 h for killing test. During the incubation, control plates were placed at 4°C in parallel, and then the mixture were plated on SDA agar and incubated at 30°C for 48 h and the live *C. albicans* were counted.

### C. albicans Eno1 modelling

*C. albicans* Eno1 structural model was built using the automated protein structure modelling server Swiss-Model (https://swissmodel.expasy.org/). BLAST and HHblits was performed for template search in the Swiss-Model template library. The model was built based on a targeted-template alignment using ProMod3. The geometry of the resulting model was regularized using a force field [32–35].

### Statistical analysis

At least three biological replicates were performed for all experiments unless otherwise indicated. Log-rank test was used for survival data analysis. For parametric data, the two-tailed Student’s *t*-test was used for analysis of two groups and one-way analysis of variance (ANOVA) was used for analysis of multiple groups. For nonparametric data, the nonparametric *t*-test or ANOVA was used. *P* value<0.05 was considered statistically significant.

## Results

### C. albicans activates host plasminogen to facilitate invasive infection

**Figure 1.**
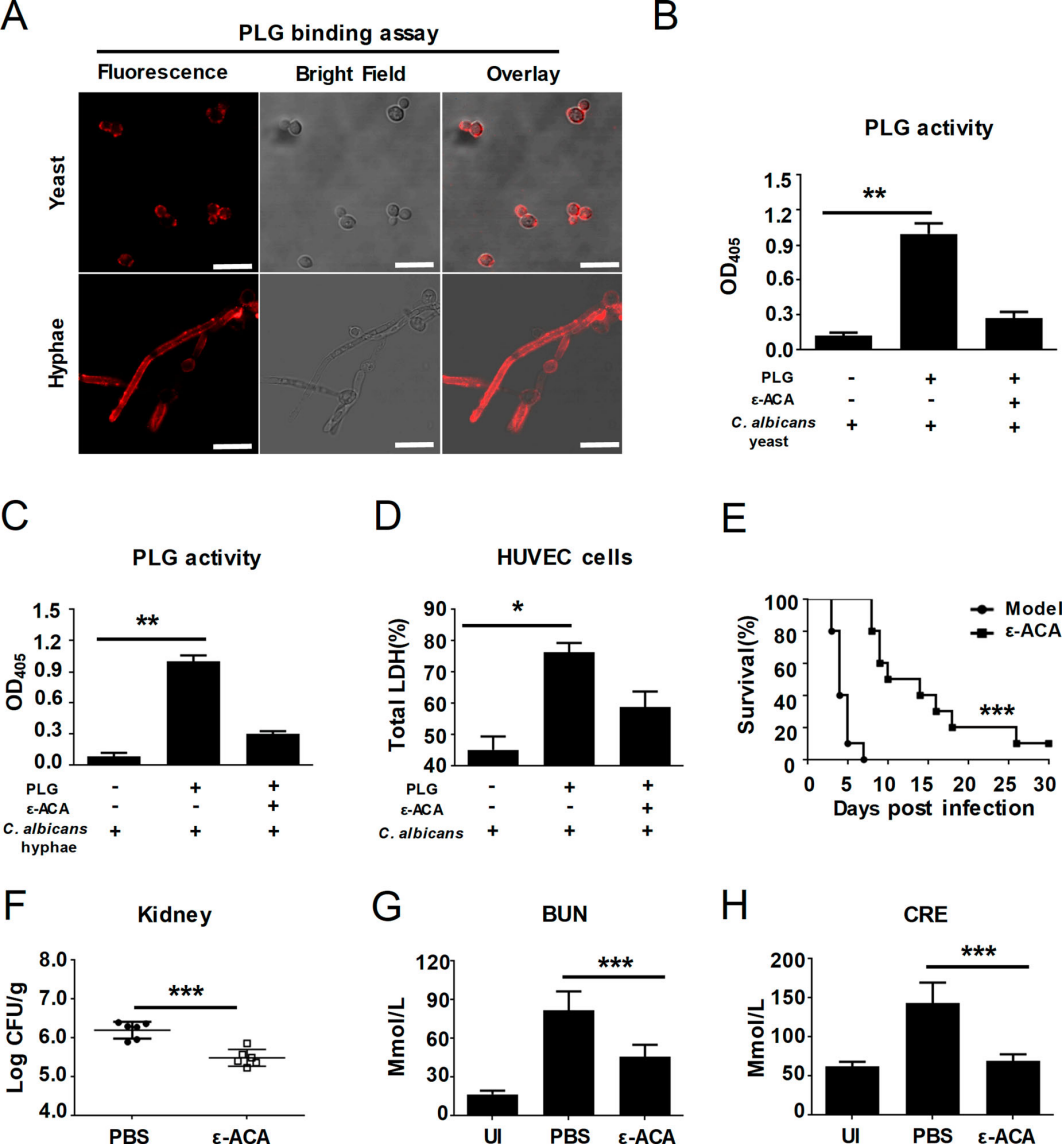
*C. albicans* activates host plasminogen to promote invasive infection. (A) Representative confocal microscope analysis of human plasminogen binding with *C. albicans* SC5314. Scale bar represents 10 μm. (B, C) Assay for yeast or hyphal form of *C. albicans* SC5314-induced plasminogen activation and the effect of plasminogen activation inhibitor ϵ-ACA (20 mM). (D) Plasminogen promoted the damage effects of *C. albicans* on HUVECs and the effect of ϵ-ACA (20 mM). HUVECs damage was determined by assaying LDH release after 12 h co-cultured with *C. albicans* SC5314 (MOI = 0.1). (E-F) C57BL/6 mice were treated ϵ-ACA (30 mg/kg) or vehicle control twice daily for 5 days after intravenously infection with *C. albicans* SC5314 (1 × 10^6^ CFU per mouse, E-H). (E) Survival of the mice was monitored for 30 days. (F) The kidney fungal burden of the mice was determined at day 2 post-infection. (G, H) Blood urea nitrogen (BUN) and creatinine (CRE) levels in mice were determined at day 2 post-infection. UI, uninfected mice; PLG, Plasminogen. Data are represented as means ± SD from triplicates of one representative experiment of three. * *P,* < 0.05; ** *P*, < 0.01; *** *P*, < 0.001 [One-way ANOVA (B, C, D, G, H); Log-rank test (E); Nonparametric *t*-test (F)].

Although previous study reported *C. albicans* could bind human plasminogen, no direct evidence for a role in establishing invasive infection was found. We first investigated that whether *C. albicans* could activate the host plasminogen system to facilitate invasive infection. We initially labelled human plasminogen with allophycocyanin 650 and observed its binding to *C. albicans* using confocal laser scanning microscopy. *C. albicans* can grow in yeast and hyphal forms, and the hyphal form has an important role in causing disease by invading epithelial cells and causing tissue damage [8]. The results revealed that plasminogen markedly bound to the surface of *C. albicans* SC5314 in both yeast and hyphae form, providing evidence that *C. albicans* may capture human plasminogen efficiently (Figure 1(A) and Supplementary Figure S1). We further found *C. albicans* could convert human plasminogen to activate plasmin, determined by assaying its proteolytic activity on the chromogenic substrate D-Val-Leu-Lys-pNA·2 HCl, and this conversion effect could be abrogated by aminocaproic acid (ϵ-ACA, a known inhibitor of plasminogen activation) (Figure 1(B,C)). Activated plasmin is a broad-specificity serine protease which could degrade several structural proteins. We found *C. albicans* could activate plasminogen to facilitate it to damage host endothelial cells, as determined by assaying LDH release after a 12-h co-culture of HUVECs with *C. albicans* (multiplicity of infection (MOI) = 0.1) in the presence of plasminogen (Figure 1(D)). To further confirm whether host plasminogen activation *in vivo* facilitates *C. albicans* infection, the mice were treated with ϵ-ACA to abolish plasminogen activity. Although ϵ-ACA has no effect on the growth of *C. albicans* (Supplementary Figure S2), our data indicated that ϵ-ACA treatment significantly improved the survival of mice infected by *C. albicans* SC5314 (Figure 1(E), 1 × 10^6^ CFU per mouse). ϵ-ACA treatment also significantly reduced kidney fungal burdens of mice infected by *C. albicans* SC5314 (Figure 1(F)). Therefore, the kidney functions of mice infected by *C. albicans* SC5314 were significantly improved after ϵ-ACA treatment, as indicated by the lower level of blood urea nitrogen (BUN) and creatinine (CRE) (Figure 1(G,H)). The above results suggested that *C. albicans* could activate host plasminogen for facilitating invasive infection.

### Eno1 plays a central role in C. albicans capturing and activating of host plasminogen

Previous studies have identified nine possible plasminogen-binding proteins on the surface of *C. albicans*, including Eno1, Tsa1, Cta1, Tdh3, Tef1, Pgk1, Adh1, Fba1, and Gpm1 [17,21]. We used a bacterial expression system to produce high purity of the above possible plasminogen-binding proteins and further compared their plasminogen binding and activating ability (Supplementary Figure S3(A), (B)). We found that *C. albicans* Eno1 displayed the strongest plasminogen binding and activating ability among these proteins (Figure 2(A,B)). Further data indicated that the *C. albicans* Eno1 bound to and activated plasminogen in a dose-dependent manner (Figure 2(C,D)). To investigate the role of Eno1 during *C. albicans* invasion of the host cell, we assayed *C. albicans* Eno1 expression during invasion of HUVECs and Caco-2 intestinal epithelial cells. The results indicated that the expression of Eno1 were significantly increased at protein level (cell wall and cytoplasmic located) (Figure 2(E,F) and (G,H)) and mRNA level (Figure 2(I,J)) (Figure 2(E,G,I) for *C. albicans* SC5314 invading HUVECs; Figure 2(F,H,J) for *C. albicans* SC5314 invading Caco-2 intestinal epithelial cells) with the infecting time increased. These findings suggested that *C. albicans* Eno1 plays a central role in capturing and activating host plasminogen to promote invasion of host cells.

**Figure 2.**
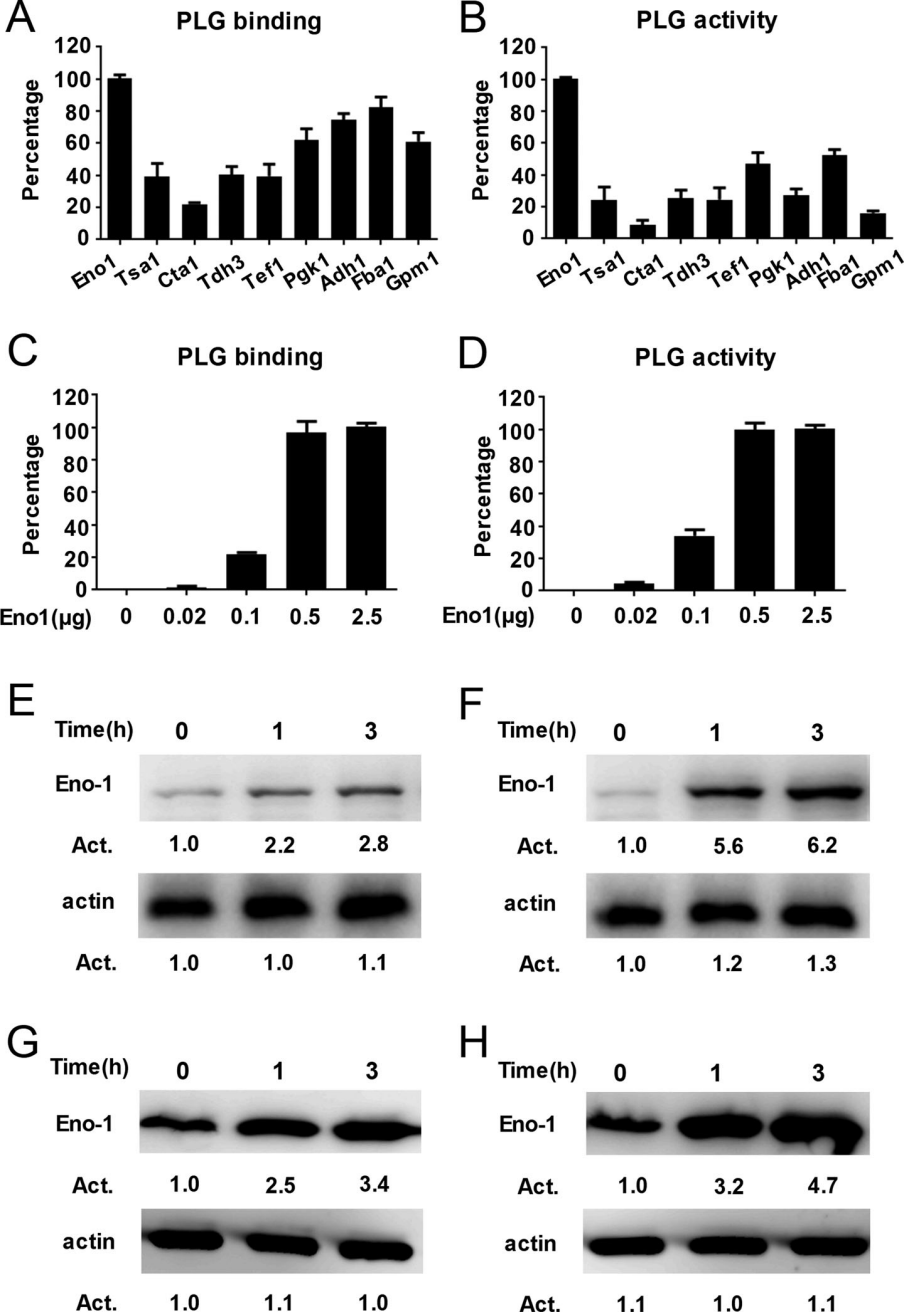
Eno1 plays a central role in *C. albicans* activating human plasminogen. (A) ELISA assays for recombinant *C. albicans* cell wall proteins binding to human plasminogen. Recombinant *C. albicans* Eno1 binding to human plasminogen was regarded as 100 percent. (B) Assays for the ability of recombinant *C. albicans* cell wall proteins to activate human plasminogen. Recombinant *C. albicans* Eno1 activating human plasminogen was regarded as 100 percent. (C) ELISA assays for recombinant *C. albicans* Eno1 binding to human plasminogen. Recombinant *C. albicans* Eno1 (2.5 μg) binding to human plasminogen was regarded as 100 percent. (D) Assays for recombinant *C. albicans* Eno1-induced activation of human plasminogen. Recombinant *C. albicans* Eno1 (2.5 μg) activating plasminogen was regarded as 100 percent. (E-J) Eno1 expression at protein level and mRNA level during *C. albicans* SC5314 invading human endothelial or epithelial cells *in vitro*. HUVECs (E, G, I) and Caco-2 intestinal epithelial cells (F, H, J) were co-cultured with *C. albicans* SC5314 for the indicated time (MOI = 1). *C. albicans* cell wall-localized (E, F) or cytoplasmic (G, H) Eno1 protein were determined by western blot and Eno1 mRNA expression levels were determined by Q-PCR (I, J). PLG, Plasminogen. Data shown in (A-D), (I) and (J) are means ± SD of triplicates from one representative experiment of three. The immunoblotting analysis shown in (E-H) are representative of three independent experiments. ** *P*, < 0.01 *** *P*, < 0.001 (One-way ANOVA).

**Figure 2.**
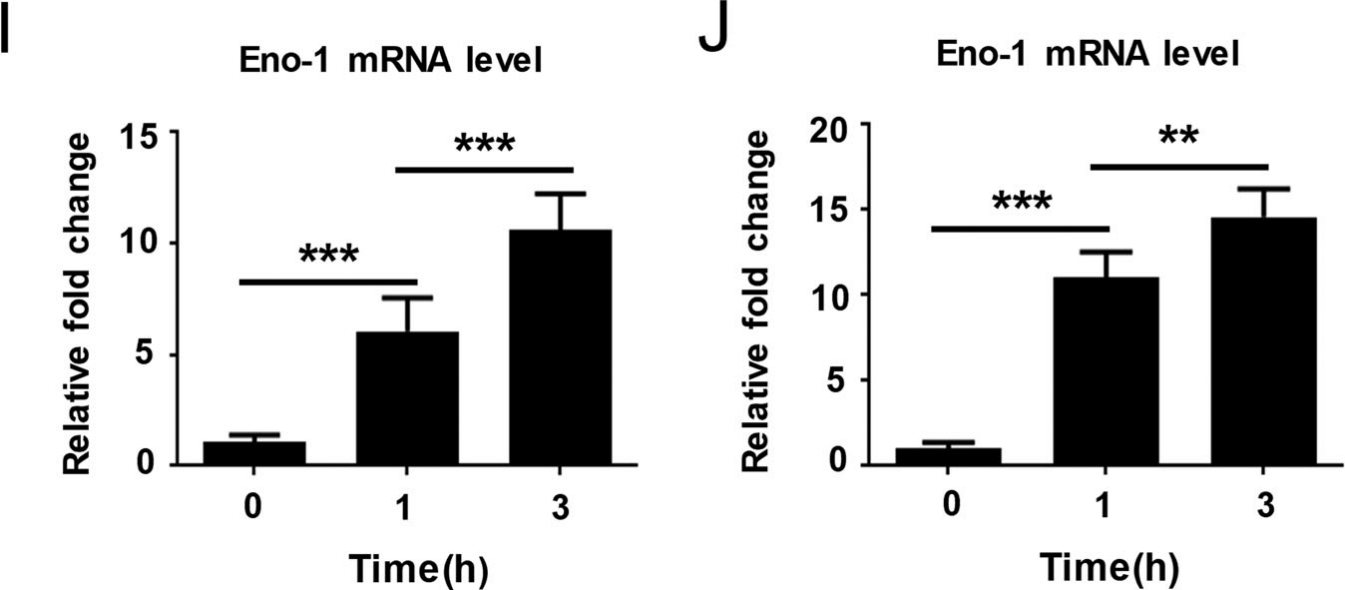
Continued.

### mAb 12D9 binds dose-dependently, specifically to C. albicans Eno1 with high affinity, and blocks C. albicans from capturing plasminogen

**Figure 3.**
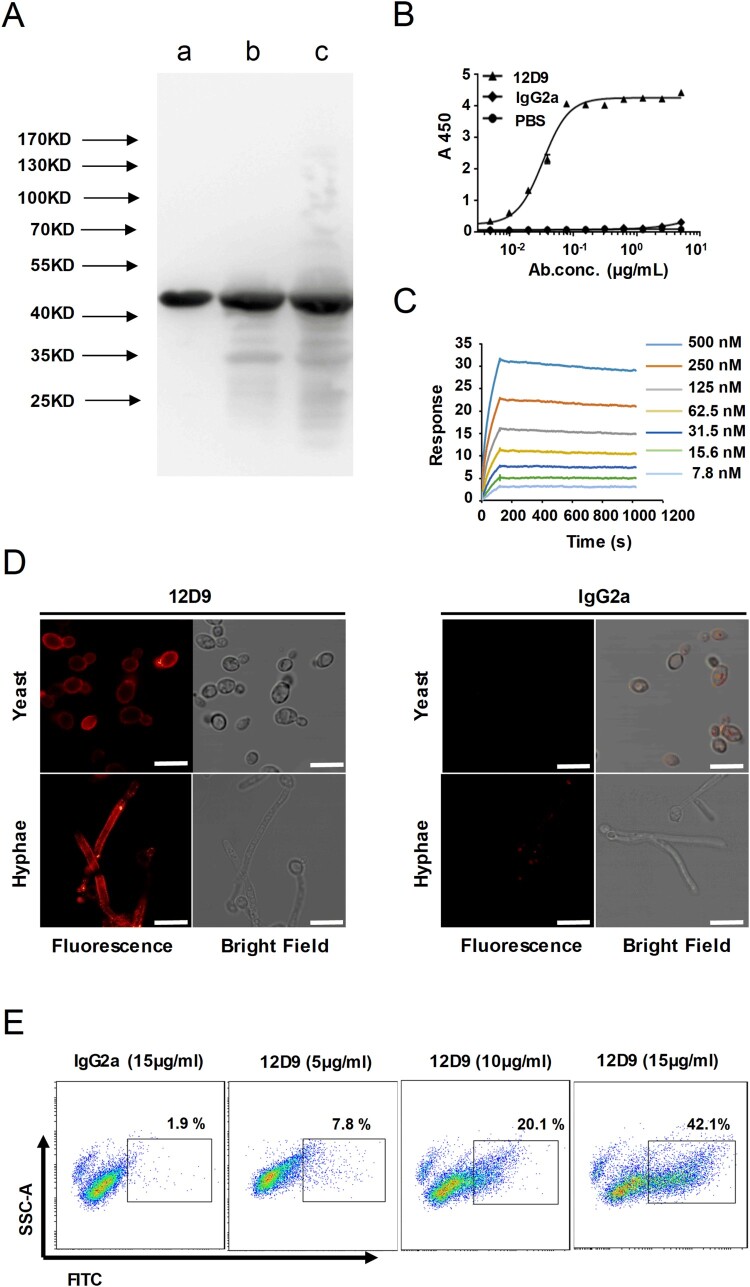
mAb 12D9 targeting *C. albicans* Eno1 displayed high affinity with recombinant Eno1 and *C. albicans* cell. (A)Western blot analysis to verify the specificity of mAb 12D9. Recombinant *C. albicans* Eno1 (Band a), cell wall extracts (Band b), and cytoplasmic extracts (Band c) from *C. albicans* SC5314 were fracted by SDS-PAGE and then subjected to immunoblotting analysis with mAb 12D9. The western bolt analysis are representative of three independent experiments. (B) ELISA assay for mAb 12D9 binding to recombinant *C. albicans* Eno1. (C) SPR analysis for interaction between the mAb 12D9 and recombinant *C. albicans* Eno1. Data shown in (B) and (C) come from one representative experiment of three. (D) Representative confocal microscope analysis for mAb 12D9 binding to *C. albicans* SC5314. Scale bar represents 10 μm. (E) Representative images analysed by flow cytometry for detection of mAb 12D9 binding to *C. albicans*. Data shown in (D, E) are representative images of three experiments.

Anti-Eno1 mAbs were generated in BALB/c mice which were immunized with recombinant *C. albicans* Eno1. Hybridomas were generated from the splenocytes of the immunized mice and P3X63Ag8.653 myeloma cells. Initially, >200 hybridoma supernatants were detected to contain IgGs which could bind with recombinant *C. albicans* Eno1 by ELISA. The hybridoma supernatants with significant affinity to *C. albicans* Eno1 were screened for ability to inhibit *C. albicans* Eno1 binding and activating of human plasminogen; whereby the pool of hybridomas expressing functional IgGs was reduced to 11. mAb 12D9 was selected from the 11 anti-Eno1 mAb based on its blocking the recombinant *C. albicans* Eno1 function of binding to plasminogen (Supplementary Figure S4). The results of western blot indicated that mAb 12D9 could specifically bind with Eno1 protein from *C. albicans* cytoplasm and cell wall (Figure 3(A)). The kinetics of mAb 12D9 binding to recombinant *C. albicans* or non-*albicans Candida* spp. Eno1 was determined using ELISA (EC50 = 0.03–0.07 μg/ml) (Figure 3(B) and Supplementary Figure S5) and SPR analysis (Figure 3(C)). The SPR analysis indicated mAb 12D9 had a high affinity with recombinant *C. albicans* Eno1 [association rate constant (ka), 4.34 × 10^4^ (1/M/s); dissociation rate constant (kd), 7.44 × 10^−5^ (1/s)]. And the estimated K_D_ (equilibrium dissociation constant) for mAb 12D9 binding to recombinant *C. albicans* Eno1 was 1.711 nM. A therapeutic delivery of mAb must be able to bind its target cells. Therefore, mAb 12D9 binding to *C. albicans* was assessed by confocal microscopy and flow cytometry. Confocal microscopy revealed that mAb 12D9 bound to the yeast and hyphae surface of *C. albicans* markedly (Figure 3(D)) and flow cytometry analysis indicated this binding was mAb 12D9 dose-dependent (Figure 3(E)). Taken together, our data indicate that mAb 12D9 displays a high affinity with recombinant *C. albicans* Eno1 and could prevent *C. albicans* from capturing plasminogen.

**Figure 4.**
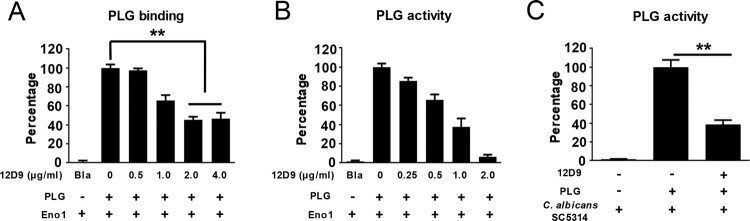
mAb 12D9 could block *C. albicans* capturing and activating human plasminogen. The assay for *C. albicans* Eno1 binding to (A) and activating (B) human plasminogen in the presence of the indicated concentration of mAb 12D9. Recombinant *C. albicans* Eno1 binding to and activating plasminogen in the absence of mAb 12D9 were regarded as 100 percent. (C) The assays for *C. albicans* SC5314 activating human plasminogen in the presence of mAb 12D9 (10μg). PLG, Plasminogen. Bla, Blank. ** *P*, < 0.01[One-way ANOVA (A, C)].

Furthermore, we found that mAb 12D9 could not only block recombinant *C. albicans* Eno1 binding and activating of plasminogen (Figure 4(A,B)), but also block the plasminogen activating ability of *C. albicans* SC5314, clinical isolate *C. albicans* Y0109 and *non-albicans Candida spp.* (Figure 4(C), Supplementary Figures S6 and S7).

### mAb 12D9 blocks C. albicans capturing plasminogen in mice to reduce invasive infection

**Figure 5.**
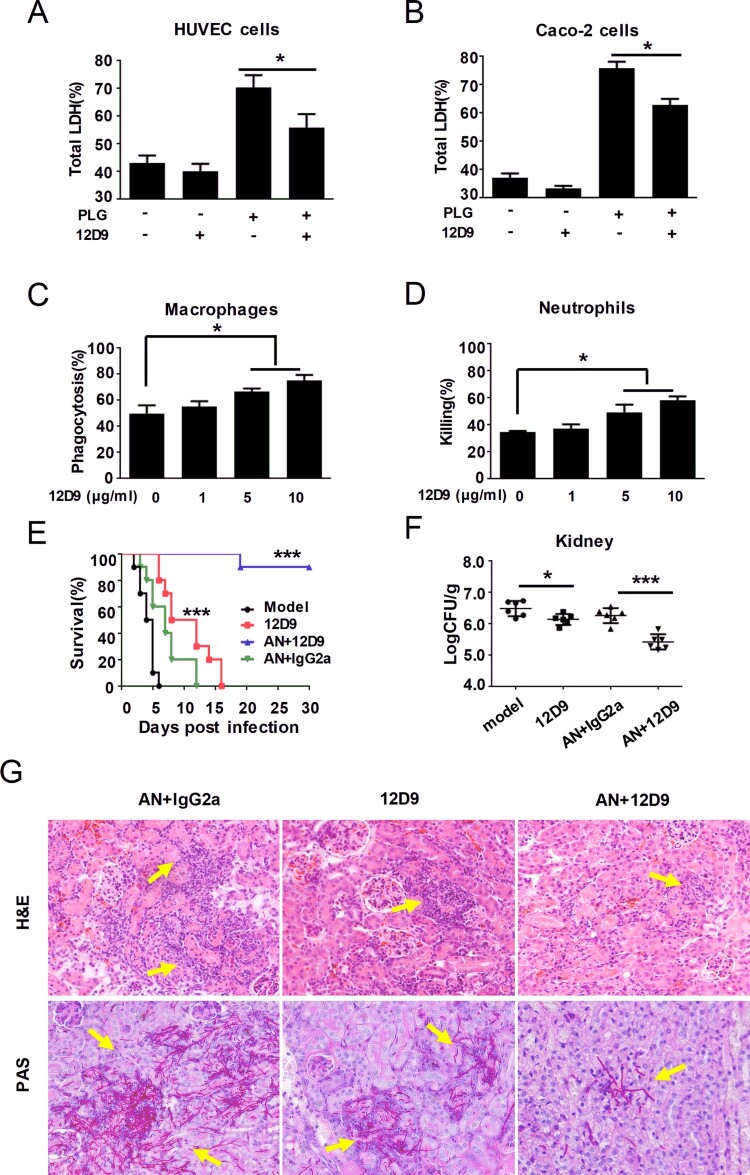
mAb 12D9 blocks *C. albicans* developing invasive infection. (A, B) HUVECs (A) and Caco-2 intestinal epithelial cells (B) damage induced by *C. albicans* in the presence of plasminogen and mAb 12D9 (10 μg/mL) was determined by assaying LDH release. Relative levels of LDH release from HUVECs or Caco-2 intestinal epithelial cells were measured after 6 h of co-culture with *C. albicans* (MOI = 0.1). (C) Phagocytosis of *C. albicans* by thioglycollate-elicited peritoneal macrophages. *C. albicans* SC5314 was co-cultured with peritoneal macrophages and the indicated concentration of mAb 12D9 at 37°C for 1 h (MOI = 0.4). The suspension was then plated on SDA agar for 48 h, after which *C. albicans* colonies were counted and the phagocytosis percent was calculated. (D) Killing of *C. albicans* by thioglycollate-elicited peritoneal neutrophil. *C. albicans* SC5314 cells were co-cultured with peritoneal neutrophils and the indicated concentration of mAb 12D9 at 37°C for 1 h. The suspension was then plated on SDA agar for 48 h, after which *C. albicans* colonies were counted and the killing percent was calculated. Data in (A-D) are represented as means ± SD from triplicates of one representative experiment of three. (E-G) C57BL/6 mice were infected with 1 × 10^6^ CFU of *C. albicans* SC5314 and treated with mAb 12D9 (30 mg/kg) and/or anidulafungin (AN) (0.1 mg/kg) via the lateral tail vein. (E) Survival of mice was monitored for 30 days (*n *= 10 per group). (F) Quantification of the fungal burden in the kidneys of mice (*n* = 6 per group) at day 2 post-infection. (G) Representative H&E (for determining inflammatory cell influx and the extent of tissue necrosis) and PAS (for *C. albicans*) staining of kidneys from infected mice with the indicated treatment at day 2 post-infection. Arrows indicate inflammatory cells influx and tissue necrosis (H&E staining) and *C. albicans* filaments in the tissues (PAS staining). Magnification 200×. Data in (G-J) are representative of three independent experiments. *, *P* < 0.05; ***, *P* < 0.001[One-way ANOVA (A-D); Log-rank test (E); Nonparametric One-way ANOVA (F)].

Damage of epithelial or endothelial cells is the key step of *C. albicans* developing invasive infection. Therefore, to investigate whether mAb 12D9 could inhibit *C. albicans* induced infection, we first tested the effect of mAb 12D9 on *C. albicans* damage of HUVECs and Caco-2 intestinal epithelial cells. In the presence of plasminogen, we found that mAb 12D9 could significantly reduce *C. albicans* medicated damage (diminished LDH release) on HUVECs and Caco-2 cells (Figure 5(A,B) and Supplementary Figure S8). While, the inhibitory effect was not observed in the absence of plasminogen with a dose-dependent manner (Figure 5(A,B)). Neutrophils and macrophages are the primary leukocytes responsible for phagocytosis and killing of invasive fungal cells. Antibody binding to pathogens can induce host innate immune cell-mediated phagocytosis and killing. mAb 12D9 mediated fungal opsono-phagocytosis and killing were measured by co-culture of *C. albicans* and macrophages or neutrophils in the presence of mAb 12D9. We found that mAb 12D9 exhibited dose-dependent opsono-phagocytosis and killing activity when macrophages and neutrophils were challenged by *C. albicans* SC5314 (Figure 5(C,D)).

To determine the antifungal infection effect of mAb 12D9 *in vivo*, mice were infected via intravenously injection of *C. albicans* SC5314 [1 × 10^6^ Colony-Forming Units (CFU) per mouse] and treated with mAb 12D9 (30 mg/kg). The data showed that mAb 12D9 could significantly improve the survival of mice (Figure 5(E)) and reduced fungal burden in the kidneys (Figure 5(F)). Additionally, mAb 12D9 also displayed significantly synergistic antifungal activity with a sub-effective dose of anidulafungin (Figure 5(F)) or fluconazole (Supplementary Figure S9(A–C)). H&E staining revealed that inflammatory influx was significantly diminished and PAS staining showed less hyphae in the kidneys of mice treated with mAb 12D9 (Figure 5(G)) (Supplementary Figure S9(D)). Furthermore, mAb 12D9 could also reduce kidney fungal burden of mice infected by clinical isolates *C. albicans* Y0109 and 0304103 (Supplementary Figure S10).

Although *C. albicans* remains the predominant fungal species that causes systemic and mucosal infections, non-*albicans Candida* spp. are also regarded as an important cause of serious candidemia [36]. Therefore, we also determined the effect of mAb 12D9 on non-*albicans Candida* spp. caused infections. We found that treatment with mAb 12D9 could significantly reduce the kidney fungal burdens in mice with candidemia caused by *Candida parapsilosis, Candida tropicalis, Candida glabrata* or *Candida krusei* (Supplementary Figure S11).

Our above results indicated that mAb 12D9 is a promising potential therapeutic antibody for both *C. albicans* and non-*albicans Candida* spp. caused invasive infections.

### Maximal activity of mAb 12D9 against C. albicans infection depends on host plasminogen activation

**Figure 6.**
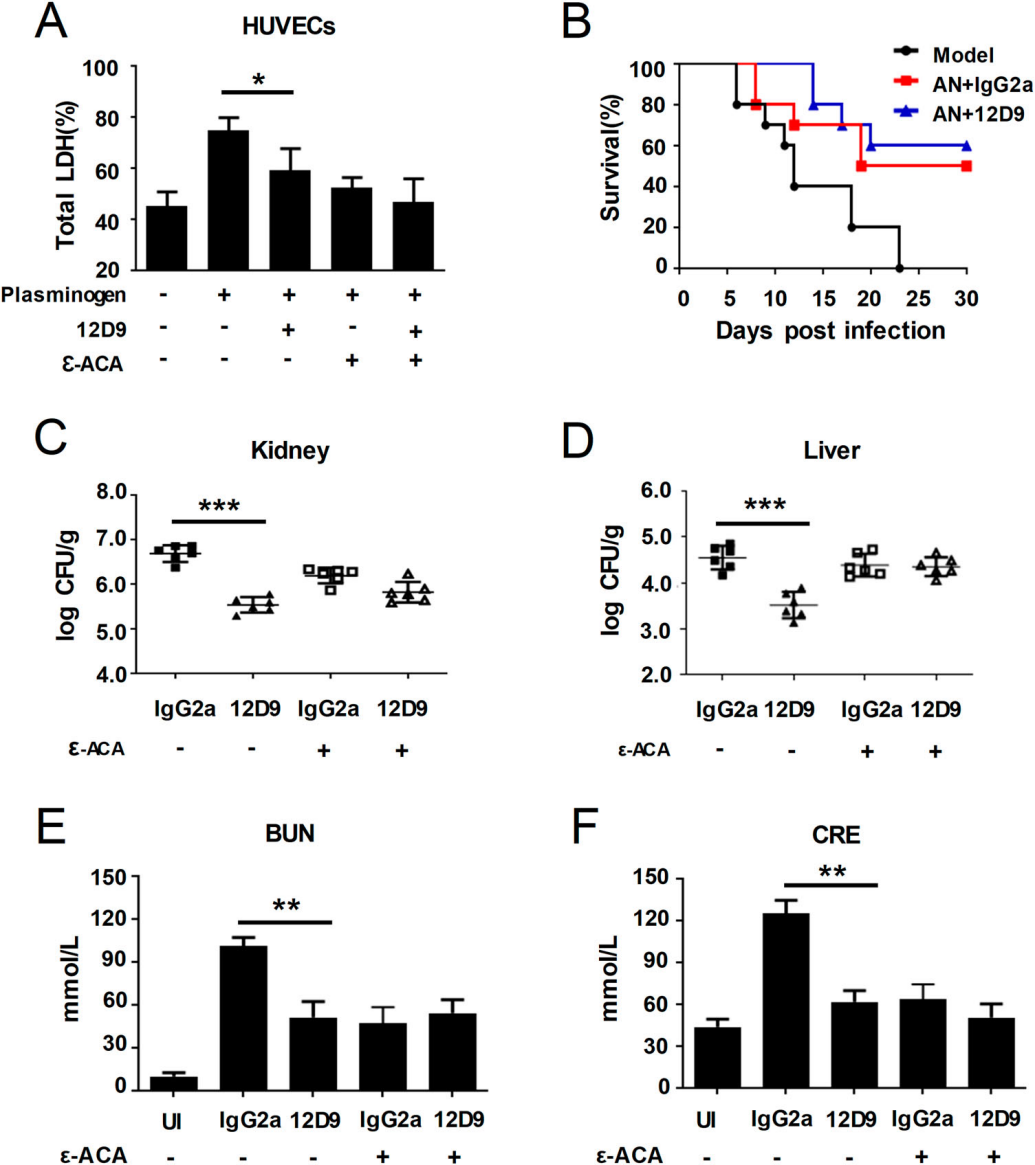
Maximal activity of mAb 12D9 against *C. albicans* infection depends on host plasminogen. (A) HUVECs damage was determined by assaying LDH release. Relative levels of LDH release from HUVECs were measured after 6 h of co-culture with *C. albicans* (MOI = 0.1) in the presence of indicated plasminogen, mAb 12D9 (4μg/mL) and ϵ-ACA (20 mM). (B-F) C57BL/6 mice were treated with ϵ-ACA (30 mg/kg) twice daily for 5 days to abolish plasminogen system after intravenously infection with *C. albicans* SC5314 (1 × 10^6^ CFU). (B) Survival of mice with the indicated mAb 12D9 (30 mg/kg) and/or anidulafungin (AN) (0.1 mg/kg) treatment was monitored for 30 days (*n *= 10 per group). (C, D) Quantification of fungal burden in kidneys (C) and liver (D) of mice treated with mAb 12D9 (30 mg/kg) and/or anidulafungin (0.1 mg/kg) (*n *= 6 per group) at day 2 post-infection. (E, F) Blood urea nitrogen (BUN) (E) and creatinine (CRE) (F) in mice were determined at day 2 post-infection. UI, uninfected mice. Data in (A-F) are representative of three independent experiments. **, *P* < 0.01; ***, *P* < 0.001; [Nonparametric One-way ANOVA (A, C-F); Log-rank test (B)].

To gain further insight into the mechanism of how mAb 12D9 against *C. albicans* infection, we examined whether the antifungal activity of mAb 12D9 is dependent on host plasminogen activation. We found that mAb 12D9 could inhibit *C. albicans* induced damage to HUVECs (determined via LDH release) in the presence of plasminogen, while the inhibit effect was abolished when the activation of plasminogen was inhibited by ϵ-ACA (Figure 6(A)). We further abolished plasminogen in mice by ϵ-ACA treatment (30 mg/kg). As a result, mAb 12D9 failed to improve survival and reduce fungal burden in the kidneys and liver of *C. albicans* infected mice with ϵ-ACA treatment (Figure 6(B–D)). In addition, no significant improvement of kidneys function (BUN and CRE) was detected in the experimental mice (Figure 6(E,F)). The above results suggested that the effect of mAb 12D9 against fungal infection depends on host plasminogen activation.

### mAb 12D9 binds to a motif in the helix 6 sheet 6 loop of C. albicans Eno1 to prevent it capturing and activating host plasminogen

**Figure 7.**
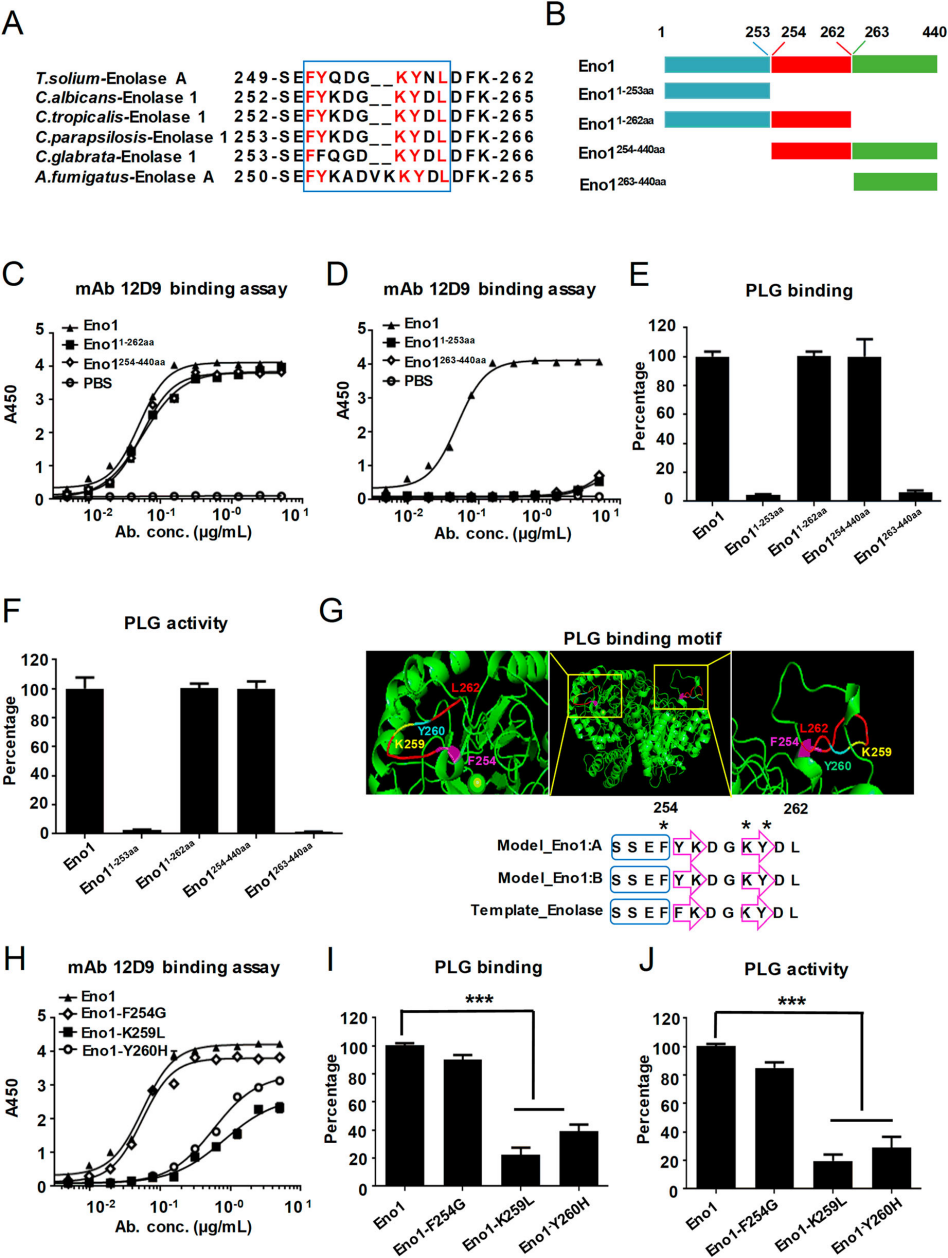
mAb 12D9 binds motif _254_FYKDGKYDL_262_ in H6S6 loop of *C. albicans* Eno1 to prevent it activating plasminogen. (A) Alignment of the H6S6 loop amino acid sequences of *T. solium* enolase A with enolase of fungal species including *C. albicans*, *C. tropicalis*, *C. parapsilosis*, *C. glabrata* and *A. fumigatus.* (B) Schematic diagram of different domains of recombinant *C. albicans* Eno1. (C, D) ELISA assay for mAb 12D9 binding to *C. albicans* Eno1 and its different domains (Eno1^254-440aa^ and Eno1^1-262aa^, C; Eno1^263-440aa^ and Eno1^1-253aa^, D). (E) Plasminogen-binding assay with different domains of recombinant *C. albicans* Eno1. (F) Plasminogen activation induced by different domains of recombinant *C. albicans* Eno1. (G) Structural model of *C. albicans* Eno1 (homo-dimer) generation using Swiss-Model. Putative Eno1-plasminogen interaction motif was marked in red, and the key plasminogen-binding residues (F254G, amaranth; K259L, yellow; Y260H, cyan) were calculated by Swiss-Model and marked with “*”. A blue box stands for α-helices and a purple arrow represents a β-strand. (H) ELISA assay for mAb 12D9 binding to recombinant *C. albicans* Eno1 and mutations (F254G, K259L and Y260H). (I) Plasminogen-binding assay for recombinant *C. albicans* Eno1 and mutations (F254G, K259L and Y260H). (J) Plasminogen activation induced by recombinant *C. albicans* Eno1 and mutations (F254G, K259L and Y260H). Recombinant *C. albicans* Eno1 binding to and activating plasminogen in the absence of mAb 12D9 were regarded as 100 percent. PLG, Plasminogen. Data in (C-F and H-J) are representative of three independent experiments. *** *P*, < 0.001 (One-way ANOVA).

The _251_FYQDGKYNL_259_ sequence in the helix 6 sheet 6 (H6S6) loop of *T. solium* EnoA has been identified as the putative plasminogen binding motif [37]. We aligned the putative plasminogen-binding loop of *T. solium* EnoA with enolase of fungal species including *C. albicans*, *C. tropicalis*, *C. parapsilosis*, *C. glabrata* and *A. fumigatus.* High similarity of plasminogen-binding motif was identified among these enolase sequences (Figure 7(A)). Specifically, the _254_FYKDGKYDL_262_ motif in the H6S6 loop of *C. albicans* Eno1 shared high sequence identity with the putative plasminogen-binding loop motif of *T. solium* EnoA (Figure 7(A)). In order to map the plasminogen-binding sites of *C. albicans* Eno1 and the antigen epitope of mAb 12D9 binding to, four domains of Eno1 were recombinant expressed: domain 1-253aa, domain 1-262aa, domain 254-440aa and domain 263-440aa (Figure 7(B) and Supplementary Figure S3(C)). We subsequently investigated the affinity of mAb 12D9 for each of the four domains of *C. albicans* Eno1. The data indicated that mAb 12D9 had a similar affinity for *C. albicans* Eno1^1-262aa^ and Eno1^254-440aa^, as well as *C. albicans* Eno1. However, mAb 12D9 exhibited low affinity for *C. albicans* Eno1^1-253aa^ and Eno1^263-440aa^ (Figure 7(C,D)). Furthermore, *C. albicans* Eno1^1-262aa^ and Eno1^263-440^ could bind and activate human plasminogen, while *C. albicans* Eno1^1-253aa^ and Eno1^263-440aa^ could not bind and activate human plasminogen (Figure 7(E,F)). The above data suggested mAb 12D9 targets to motif _254_FYKDGKYDL_262_ in the H6S6 loop of *C. albicans* Eno1 to block it binding to and activating plasminogen. Three-dimensional structural model showed that residues F254, K259, and Y260 plays critical role for maintain the structure of motif _254_FYKDGKYDL_262_ (Figure 7(G)). Based on this, we recombinant expressed three mutants of *C. albicans* Eno1(F254G, K259L and Y260H). We found that mAb 12D9 displayed decreased affinity with the Eno1-K259L and Eno1-Y260H mutants but had a similar affinity for the Eno1-F254G mutant compared to *C. albicans* Eno1 (Figure 7(H)). Furthermore, the Eno1-K259L and Eno1-Y260H mutants exhibited significantly lower plasminogen binding and plasminogen activating abilities, while similar levels of plasminogen binding and activation of the Eno1-F254G mutant were detected compared to *C. albicans* Eno1 (Figure 7(I,J)). Our results revealed that mAb 12D9 binds to a motif (_254_FYKDGKYDL_262_) with key residues of K259 and Y260 in the H6S6 loop of *C. albicans* Eno1 to prevent it capturing and activating host plasminogen.

## Discussion

Physical barriers play an important role in host controlling dissemination of pathogens. Invasion and damaging host physical barriers are the premise of *C. albicans* progressing from colonization to systemic infection. Previous study suggested that Eno1 is involved in *C.albicans* colonization and invasion of host cells barriers *in vitro* [27,28]. Our findings demonstrated that the *C. albicans* cell wall-localized Eno1 can capture and manipulate host plasminogen system to damage mucosal epithelium and vascular endothelial cells. We further discovered that mAb 12D9 which targets to Eno1 could prevent host plasminogen being captured by *C. albicans* and was effective for controlling candidemia *in vivo*.

The mammalian plasminogen-plasmin system plays a central role in fibrinolysis, ECM degradation, and cell migration [13]. Previous studies have suggested that pathogens such as *B. burgdorferi* and *S. aureus* can activate host plasminogen system to degrade the ECM of mucosal epithelium for promoting invasive infections [18,38]. Here, we found that *C. albicans* can efficiently capture and activate of human plasminogen system (Figure 1(A–C) and Supplementary Figure S1). Since *C. albicans* colonizes human mucosal surfaces, its ability to bind to and activate plasminogen may be an important pathogenic mechanism. We found that plasminogen binding makes *C. albicans* more virulent in the HUVECs-*C. albicans* interaction model *in vitro* (Figure 1(D)).

Previous work has demonstrated that ϵ-ACA can effectively inhibit activity of plasminogen [39]. Therefore, we abolished plasminogen-plasmin system using ϵ-ACA in mice to explore the role of plasminogen activation in *C. albicans* caused systemic infection *in vivo*. Our results demonstrated that ϵ-ACA could lessen *C. albicans* systemic infection in mice and highlight an important role of plasminogen activation in *C. albicans* establishing systemic infection (Figure 1(E–H)).

Enolase catalyses the conversion of 2-PGA to PEPE and its activity is critical for both glycolysis and gluconeogenesis across virtually all taxa. However, it has been reported that enolase is also cell wall-localized as a multifunctional protein with non-glycolytic functions in several pathogens [21–23]. α-Enolase has been identified as a primary plasminogen receptor on the surface of *streptococcal* groups [21,40–42]. Eno1 was responsible for transglutaminase activity on cell wall of *C. albicans* and acts as the major antigen in patients with candidiasis. Both passive and active vaccinations with *C. albicans* Eno1 display protective effect against disseminated candidiasis [43,44]. The role of enolase in pathogenicity of a variety of fungal species, including *Aspergillus nidulans*, *Aspergillus fumigatus*, *C. albicans* and *Paracoccidioides brasiliensis* has been reported [21,45,46]. *C. albicans* Eno1 null mutant exhibits abnormal hyphal formation, attenuated virulence and is more susceptible to antifungal agents such as amphotericin B and azoles [29]. In this study, we found that Eno1 displayed the strongest plasminogen-binding and activation activity in the nine possible plasminogen receptors of *C. albicans* (Figure 2(A–D)). Our finding demonstrated that Eno1 expression was significantly increased during *C. albicans* invading host cell (Figure 2(E–J)). Hence, we propose that Eno1 plays the most important role in pathogenicity of *C. albicans* by activating host plasminogen. However, Tef1 and Adh1 also displayed plasminogen binding and activating ability which should not be neglected (Figure 2(A,B)), and it will be investigated in our future study.

Neutralization is the most direct mechanism by which antibodies counteract pathogens. The Fc domain of antibodies can also recruit innate immune cells to engage in effector functions such as opsono-phagocytosis and cytotoxicity [47]. Our study identified and characterized mAb 12D9 with high affinity to *C. albicans* and non-*albicans Candida* spp. Eno1 (Figure 3(A–C) and Supplementary Figure S5). We found that mAb 12D9 could markedly bind to *C. albicans* and block it capturing and activating plasminogen (Figure 3(D,E), Figure 4 and Supplementary Figure S6). Since *C. albicans*-mediated activation of host plasminogen could promote invasive infections, we investigated the anti-infective effect of mAb 12D9 using an *in vitro* host cell-*C. albicans* interaction model. We found that mAb 12D9 could attenuate the damage of *C. albicans* on endothelial or epithelial cells (Figure 5(A–D)), as well as promote recruitment of macrophages and neutrophils to target *C. albicans* via its Fc domain (Figure 5(E,F)). As mAb 12D9 exerts both Eno1 blocking and opsono-phagocytic effects, we further confirmed these *in vitro* findings using a systemic infection mouse model. The experiments indicated that mAb 12D9 monotherapy or combination therapy with anidulafungin or fluconazole displayed significant antifungal activity in mice (Figure 5(E–G)) (Supplementary Figure S9). Since enolase is a highly conserved protein across *Candida* spp. It is reasonable that mAb 12D9 also has antifungal effects in mice with non-*albicans Candida* spp. caused invasive infection (Supplementary Figure S11). Together, our data suggested that mAb 12D9 may be a potential agent for invasive *Candida* spp. infections.

Blasting peptide sequence in *Candida* Genome Database (http://www.candidagenome.org) demonstrated that Eno1 is the only protein in *C. albicans* containing FYKDGKYDL sequence. Alignment analysis revealed that the _254_FYKDGKYDL_262_ motif in *C. albicans* Eno1 shared high sequence identity with a putative plasminogen-binding motif of *T. solium* Enolase A (_251_FYQDGKYNL_259_) (Figure 7(A,B)). By testing the affinity of mAb 12D9 and various recombinant *C. albicans* Eno1 domains, we confirmed that _254_FYKDGKYDL_262_ motif in *C. albicans* Eno1 was the antigen epitope of mAb 12D9 (Figure 7(B–D)). Our data further indicated that the motif _254_FYKDGKYDL_262_ is also required for *C. albicans* Eno1 binding and activation of plasminogen (Figure 7(E,F)). To date, the crystal structure of *C. albicans* Eno1 has not been reported. It is classified by the CATH database as a member of the enolase superfamily with the code 3.20.20.120, which indicates that the *C. albicans* Eno1 proteins consists mainly of α-helices and β-strands and has a TIM-type α-β-barrel. Swiss-Model analysis revealed that the motif _254_FYKDGKYDL_262_ is located in the H6S6 loop of *C. albicans* Eno1, and suggested that residue F254 in α-helix 6 and residues K259 and Y260 in β-sheet 6 were important for the secondary structure of this domain (Figure 7(G)). The analysis interaction of Eno1 F254G, K259L and Y260H mutants with human plasminogen confirmed that residues K259 and Y260 are key for Eno1 induced human plasminogen activation (Figure 7(G)). Affinity analysis indicated that Eno1 K259 and Y260 were required for mAb 12D9 binding (Figure 7(H–J)).

Invasive candidiasis is an important health-care-associated fungal infection and is widely recognized as a major cause of morbidity and mortality in the health-care environment despite antifungal therapy. Our present study identified that *C. albicans* have the ability to capture and “subvert” host plasminogen system to facilitate tissue invasion, and indicated that cell wall-localized Eno1 plays a central role in this process. Base on this finding, we further characterized the mAb 12D9 with high affinity to the _254_FYKDGKYDL_262_ motif in H6S6 loop of *C. albicans* Eno1. mAb 12D9 showed its ability to prevent host plasminogen being captured and “subverted” by *C. albicans*, providing a potential novel treatment strategy for invasive fungal infections.

## Supplementary Material

Table_S2.docx

Table_S1.docx

Figure_S11.docx

Figure_S10.docx

Figure_S9.docx

Figure_S8.docx

Figure_S7.docx

Figure_S6.docx

Figure_S5.docx

Figure_S4.docx

Figure_S3.docx

Figure_S2.docx

Figure_S1.docx
