# Supplementary material for: Preventing Candida albicans from subverting host plasminogen for invasive infection treatment
Source: Emerg Microbes Infect. 2020 Nov 3;9(1):2417–32. doi: 10.1080/22221751.2020.1840927 (PMC7646593; doi:10.1080/22221751.2020.1840927)
Supplement: Table_S2.docx [file TEMI_A_1840927_SM4536.docx]

| **Oligonucleotides** | |
| --- | --- |
| Eno1-F | GGATCCATGTCTTACGCCACTAAAATC |
| Eno1-R | CTCGAGTTACAATTGAGAAGCCTTT |
| Eno1^1-253aa^-F | GGATCCATGTCTTACGCCACTAAAATC |
| Eno1^1-253aa^ -R | CTCGAGTTCAGATGAAGCAACATCCATG |
| Eno1^1-262aa^-F | GGATCCATGTCTTACGCCACTAAAATC |
| Eno1^1-262aa^-R | CTCGAGCAAGTCGTATTTACCGTCCTTGTAGA |
| Eno1^254-440aa^-F | GGATCCTTCTACAAGGACGGTAAATACG |
| Eno1^254-440aa^-R | CTCGAGTTACAATTGAGAAGCCTTT |
| Eno1^263-440aa^-F | GGATCCAATCCGACCCATCTAAATGGTT |
| Eno1^263-440aa^-R | CTCGAGTTACAATTGAGAAGCCTTT |
| Tsa1-F | GGATCCATGGCTCCAGTCGTTCAA |
| Tsa1-R | CTCGAGTTATTTGTTGACTTTGTT |
| Cta1-F | GGATCCATGGCTCCAACATTTACGA |
| Cta1-R | CTCGAGTTATTTTCTTGGAGATAA |
| Tdh3-F | GGATCCATGGCTATTAAAATTGGTAT |
| Tdh3-R | CTCGAGTCAAGCAGAAGCTTTAG |
| Tef1-F | GGATCCATGGGTAAAGAAAAAACT |
| Tef1-R | CTCGAGTTATTTCTTAGCAGCTTTT |
| Pgk1-F | GGATCCATGTCATTATCTAACAAAT |
| Pgk1-R | CTCGAGTTAGTTTTTGTTGGAAA |
| Adh1-F | GGATCCATGTCTGAACAAATCC |
| Adh1-R | CTCGAGTTATTTACTGGTGTCCAA |
| Fba1-F | GGATCCATGGCTCCTCCAGCAGT |
| Fba1-R | CTCGAGTTACAATTGTCCTTTAGT |
| Gpm1-F | GGATCCATGCCAAAGTTAGTTTT |
| Gpm1-R | CTCGAGGCTCAAGGTCAAAAGAA |

**TABLE S2.** Primers used for amplifying the gene encoding

*C. albicans* cell wall proteins.
