# Supplementary material for: Preventing Candida albicans from subverting host plasminogen for invasive infection treatment
Source: Emerg Microbes Infect. 2020 Nov 3;9(1):2417–32. doi: 10.1080/22221751.2020.1840927 (PMC7646593; doi:10.1080/22221751.2020.1840927)
Supplement: Table_S1.docx [file TEMI_A_1840927_SM4535.docx]

**TABLE S1.** Primers used in q-PCR.

| **Oligonucleotides** | |
| --- | --- |
| GAPDH-F | AAGAAGGTGGTGAAGCAGGC |
| GAPDH-R | CTCGAGTTACAATTGAGAAGCCTTT |
| Eno1-F | AAGGCATTCCATTGTACAAA |
| Eno1-R | TCAAGTTGTGGTAAACTTCTG |
