# Supplementary material for: Preventing Candida albicans from subverting host plasminogen for invasive infection treatment
Source: Emerg Microbes Infect. 2020 Nov 3;9(1):2417–32. doi: 10.1080/22221751.2020.1840927 (PMC7646593; doi:10.1080/22221751.2020.1840927)
Supplement: Figure_S11.docx [file TEMI_A_1840927_SM4534.docx]

**FIG S11 mAb 12D9 blocks non-*albicans Candida* spp. developing invasive infection.** C57BL/6 mice were intravenously infected with *C. parapsilosis* ATCC34136 (1×10^6^ CFU per mice) (A), *C. tropicalis* ATCC20026 (1×10^6^ CFU per mice) (B), *C. glabrata* ATCC28226 (1×10^7^ CFU per mouse) (C), *C. krusei* ATCC6258 (5×10^5^ CFU per mice) (D) and treated with mAb 12D9 (30 mg/kg) and/or anidulafungin (AN) (0.1mg/kg) via the lateral tail vein. Quantification of the fungal burden in kidneys at day 2 post-infection. Data are representative of three independent experiments. ***** *P*, < 0.05; ****** *P*, < 0.01 ******* *P*, < 0.001 (One-way ANOVA).
