# Supplementary material for: Preventing Candida albicans from subverting host plasminogen for invasive infection treatment
Source: Emerg Microbes Infect. 2020 Nov 3;9(1):2417–32. doi: 10.1080/22221751.2020.1840927 (PMC7646593; doi:10.1080/22221751.2020.1840927)
Supplement: Figure_S10.docx [file TEMI_A_1840927_SM4533.docx]

**FIG S10** **mAb 12D9 blocks clinical isolates of *C. albicans* developing invasive infection.** C57BL/6 mice were intravenously infected with clinical isolates of *C. albicans* Y0109 (A) and 0304103 (B) (1×10^6^ CFU per mouse) and treated with mAb 12D9 (30 mg/kg) and/or anidulafungin (AN) (0.1mg/kg) via the lateral tail vein. The fungal burden in kidneys were evaluated at day 2 post-infection. Data are representative of three independent experiments. *****, *P* < 0.05; *******, *P* < 0.001 (Nonparametric One-way ANOVA).
