# Supplementary material for: Preventing Candida albicans from subverting host plasminogen for invasive infection treatment
Source: Emerg Microbes Infect. 2020 Nov 3;9(1):2417–32. doi: 10.1080/22221751.2020.1840927 (PMC7646593; doi:10.1080/22221751.2020.1840927)
Supplement: Figure_S9.docx [file TEMI_A_1840927_SM4532.docx]

**

**

**FIG S9 mAb 12D9 exhibits synergistic antifungal activity against *C. albicans* infection with fluconazole.** C57BL/6 mice were intravenously infected with 1×10^6^ CFU of *C. albicans* SC5314 and treated with mAb 12D9 (30 mg/kg) and/or fluconazole (FLC) (0.1mg/kg) via lateral tail vein. (A) Survival of mice was monitored for 30 days. (B, C) The kidney (B) and liver (C) fungal burdens of mice with indicated treatment (n = 6 per group) at day 2 post-infection. (D) Representative H&E (for the inflammatory cells influx and the extent of tissue necrosis) and PAS (for *C. albicans*) staining of kidneys from infected mice with indicated treatment at day 2 post-infection. Arrows indicate inflammatory cells influx and tissue necrosis (H&E staining) and *C. albicans* filaments in the tissues (PAS staining). Magnification = 200 ×. Data in (A-D) are representative of three independent experiments. ******* *P*, < 0.001[Log-rank test (A); Nonparametric One-way ANOVA (B, C)].
