# Supplementary material for: Preventing Candida albicans from subverting host plasminogen for invasive infection treatment
Source: Emerg Microbes Infect. 2020 Nov 3;9(1):2417–32. doi: 10.1080/22221751.2020.1840927 (PMC7646593; doi:10.1080/22221751.2020.1840927)
Supplement: Figure_S8.docx [file TEMI_A_1840927_SM4531.docx]

**FIG S8 Damage of HUVECs (A) and Caco-2 intestinal epithelial cells (B) induced by *C. albicans* in the presence of plasminogen and mAb 12D9 (1, 5μg/ml) was determined by assaying LDH release.** Relative levels of LDH release from HUVECs or Caco-2 intestinal epithelial cells were measured after a 6-h co-culture with *C. albicans* (MOI = 0.1).
