# Supplementary material for: Preventing Candida albicans from subverting host plasminogen for invasive infection treatment
Source: Emerg Microbes Infect. 2020 Nov 3;9(1):2417–32. doi: 10.1080/22221751.2020.1840927 (PMC7646593; doi:10.1080/22221751.2020.1840927)
Supplement: Figure_S7.docx [file TEMI_A_1840927_SM4530.docx]

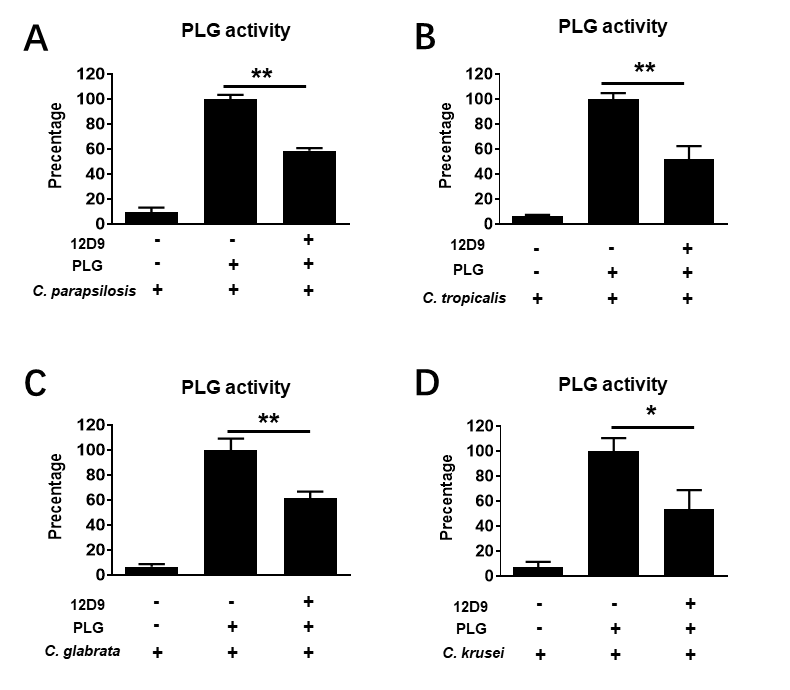


**FIG S7 mAb 12D9 could block non-*albicans Candida* spp. activating human plasminogen.** Human plasminogen activation in the presence of mAb 12D9 (10μg) by *C. parapsilosis* ATCC 34136 (A), *C. tropical* ATCC 20026 (B), *C. glabrata* ATCC 28226 (C), *C. krusei* ATCC 6258 (D). Non-*albicans Candida* spp. activating plasminogen in the absence of mAb 12D9 were regarded as 100 percent. PLG, Plasminogen. ***** *P*, < 0.05, ****** *P*, < 0.01(One-way ANOVA).
