# Supplementary material for: Preventing Candida albicans from subverting host plasminogen for invasive infection treatment
Source: Emerg Microbes Infect. 2020 Nov 3;9(1):2417–32. doi: 10.1080/22221751.2020.1840927 (PMC7646593; doi:10.1080/22221751.2020.1840927)
Supplement: Figure_S6.docx [file TEMI_A_1840927_SM4529.docx]

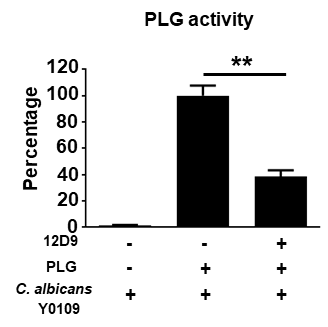


**FIG S6 mAb 12D9 inhibited clinical isolated *C. albicans* Y0109 activating human plasminogen (10μg).** *C. albicans* Y0109 activating plasminogen in the absence of mAb 12D9 were regarded as 100 percent. PLG, Plasminogen. Data shown are representative images of three experiments.
