# Supplementary material for: Preventing Candida albicans from subverting host plasminogen for invasive infection treatment
Source: Emerg Microbes Infect. 2020 Nov 3;9(1):2417–32. doi: 10.1080/22221751.2020.1840927 (PMC7646593; doi:10.1080/22221751.2020.1840927)
Supplement: Figure_S5.docx [file TEMI_A_1840927_SM4528.docx]

**FIG S5 ELISA assay for mAb 12D9 binding to recombinant Eno1 proteins of** **non-*albicans Candida* spp.** [*C. parapsilosis* ATCC34136 (A), *C. tropicalis* ATCC20026 (B), *C. glabrata* ATCC28226 (C) and *Candida krusei* ATCC6258 (D)].
