# Supplementary material for: Preventing Candida albicans from subverting host plasminogen for invasive infection treatment
Source: Emerg Microbes Infect. 2020 Nov 3;9(1):2417–32. doi: 10.1080/22221751.2020.1840927 (PMC7646593; doi:10.1080/22221751.2020.1840927)
Supplement: Figure_S4.docx [file TEMI_A_1840927_SM4527.docx]

**

**

**FIG S4 Blocking recombinant *C. albicans* Eno1 binding to plasminogen by murine hybridoma supernatant containing anti-Eno1 antibody.** The affinity of recombinant *C. albicans* Eno1 with plasminogen was measured in the presence of murine hybridoma supernatant containing anti-Eno1 antibody by ELISA method. Mouse-nonspecific IgG2a was regarded as 100 percent. PLG, Plasminogen. Data are representative of three independent experiments.
