# Supplementary material for: Preventing Candida albicans from subverting host plasminogen for invasive infection treatment
Source: Emerg Microbes Infect. 2020 Nov 3;9(1):2417–32. doi: 10.1080/22221751.2020.1840927 (PMC7646593; doi:10.1080/22221751.2020.1840927)
Supplement: Figure_S3.docx [file TEMI_A_1840927_SM4526.docx]

**FIG S3 SDS-PAGE of recombinant *C. albicans* proteins.** (A) Cat1, Tef1, Eno1, Pgk1, Fba1 and Tsa1. (B) Tdh3, Gpm1 and Adh1. (C) Eno1^1-262^, Eno1^1-253^_,_ Eno1^254-440^ and Eno1^263-440^.
