# Supplementary material for: Preventing Candida albicans from subverting host plasminogen for invasive infection treatment
Source: Emerg Microbes Infect. 2020 Nov 3;9(1):2417–32. doi: 10.1080/22221751.2020.1840927 (PMC7646593; doi:10.1080/22221751.2020.1840927)
Supplement: Figure_S2.docx [file TEMI_A_1840927_SM4525.docx]

**FIG S2 Ɛ-ACA has no effect on growth of *C. albicans*.** *C. albicans* SC5314 grew in YPD broth with Ɛ-ACA (20 or 60 mM) or negative control PBS buffer (initial OD_620_ was 0.1) at 30°C with 200 rpm shaking. The OD_620_ was assayed at the indicated time point. Data are representative of three independent experiments.
