# Supplementary material for: Preventing Candida albicans from subverting host plasminogen for invasive infection treatment
Source: Emerg Microbes Infect. 2020 Nov 3;9(1):2417–32. doi: 10.1080/22221751.2020.1840927 (PMC7646593; doi:10.1080/22221751.2020.1840927)
Supplement: Figure_S1.docx [file TEMI_A_1840927_SM4524.docx]

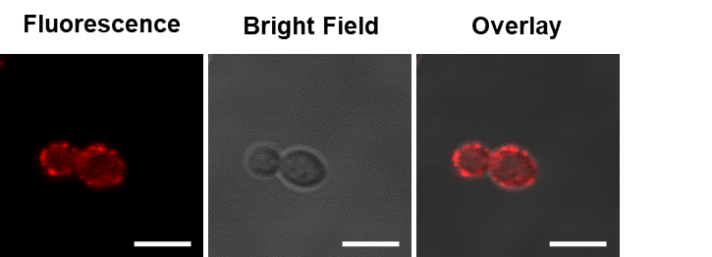


**FIG S1 Representative single**-**cell confocal microscope analysis images** **of human plasminogen binding with *C. albicans* SC5314.** Scale bar represents 4 μm.
